# The myth that Nigerians are immune to SARS-CoV-2 and that COVID-19 is a hoax are putting lives at risk

**DOI:** 10.7189/jogh.10.020375

**Published:** 2020-12

**Authors:** Oluwadamilola Aiyewumi, Malachy Ifeanyi Okeke

**Affiliations:** Department of Natural and Environmental Sciences, Biomedical Science Concentration, School of Arts and Sciences, American University of Nigeria, Yola, Adamawa State, Nigeria

Severe acute respiratory syndrome coronavirus 2 (SARS-CoV-2) is the etiological agent of coronavirus disease 2019 (COVID-19). COVID-19 virus was first reported in Wuhan China in December 2019 but has since taken a pandemic course affecting all parts of the world. As of August 2, 2020, the World Health Organization (WHO) has reported 17 660 523 cases worldwide including 680 894 deaths [1]. In Nigeria, the index COVID-19 was reported on February 27, 2020 [2]. This extension of the pandemic to Nigeria elicited an overwhelming response of panic and fear sweeping across the nation. Social media has tremendous impact on the Nigerian public’s collective perception, knowledge and attitude about COVID-19. In this case of COVID-19, it has probably done more harm by its generation of gratuitous alarm and exploitation as evidenced by the spread of fear, offer of fake cures, and the dismissal of medical advice [3]. This is further exacerbated when these misleading messages are endorsed by influential people [4]. Consequently, in an effort to quell this tide of disinformation and soothe the fears of ignorant Nigerians buried in conspiracy theories, conventional mass media coverage in news and print media has been heightened by Nigerian Centre for Disease Control (NCDC), the Federal and State governments. Despite this extensive media coverage, it may seem that Nigerians rely on these misleading sources of information rather than factual and evidence-based information from national and international competent public health authorities. This may have contributed significantly to the steady rise of confirmed cases and deaths due to COVID-19 in Nigeria (**Figure 1**). Indeed, as of July 31, 2020, Nigeria has recorded 43 151 confirmed COVID-19 cases including 879 deaths [5].

**Figure 1:**
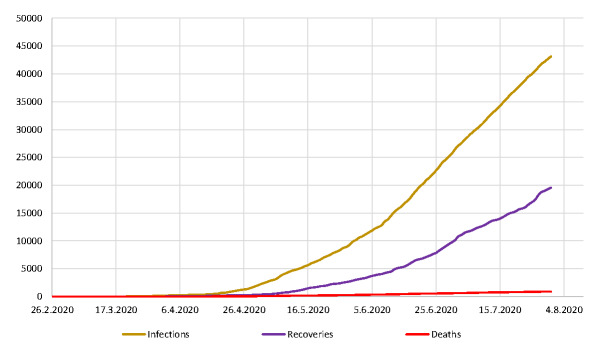
Cumulative number of infections, recoveries and deaths due to COVID-19 in Nigeria from February to July 2020. Data for the line graph was obtained from the Nigerian Centre for Disease Control (NCDC).

In this article, we aim to debunk the two most damaging myths pertaining to the COVID-19 epidemic in Nigeria, that is, that the virus is a hoax, and Nigerians are believed to be immune to the disease, highlight the negative consequences of such myths to COVID-19 mitigation and containment strategies and proffer solutions on how to curtail the spread of COVID-19 misinformation and disinformation.

## ARE NIGERIANS IMMUNE TO SARS-COV-2?

The story behind the myth of Nigerian immunity to COVID-19 stems from the mistaken belief that blacks possess an innate or adaptive protective immune response to SARS-CoV-2. This disinformation originating since the later days of January is not just in existence in Nigeria but has been circulated amongst a myriad of black communities across borders. These speculations were broadcast by various websites and blogs and featured as the butt of several gags and jokes. From their standpoint, it was viewed as a relief that for once the blacks had been excused from racial disparities that have disproportionately been unfavourable to them [6]. However, the reason for this initial inequality was not considered as obviously due to the ethnically homogenous state of China from where the novel virus was first identified. Once this myth was presented and proven to be grossly inaccurate, with an even greater number of blacks being ravaged by the virus [7], many Nigerians nonetheless believed they had many problems but the virus was not one of them. The myth had evolved to the belief that the virus could not withstand the tropical climate of the country [4]. This had since been dispelled with the seeping in of the virus into the country and the rise of infections and deaths on a daily basis.

Well, are Nigerians really immune to the virus? The answer is clearly in the negative. In fact, with the socioeconomic conditions wrecking the nation in the form of weak health care system and abject poverty, genetic predisposition to various comorbidities including ischaemic heart disease (IHD), stroke, hypertension, diabetes, HIV/AIDS burden and overpopulation, the risk is amplified [6,7]. By virtue of the characteristic disadvantages that plague the country and even Africa as a whole, the continent has been predicted to feel the brunt of the pandemic notwithstanding the comparably low number of recorded cases and deaths [8]. However, more than five months since the first index case of COVID-19 in Nigeria, the apocalyptic forecasts have not happened and both mortality and morbidity are low compared to the global trend [9]. While this is good news, it has the unintended effect of fuelling the myth of immunity to COVID-19 as many Nigerians observe that the dire predictions of exponential rise in infections, deaths and hospitalization have not materialised, hence the mistaken belief that they are immune. The Nigerian public health authority must communicate in a clear and focused manner that the milder outcome of COVID-19 in Nigeria so far is not evidence of immunity and emphasize that at the moment there is no scientific evidence that Nigerians are immune to COVID-19.

## IS COVID-19 A HOAX?

The sad truth about these present times is that any health calamity will be accompanied by an inundation of misinformation pertaining to its origin. Time and time again, a tide of conspiracy theories follow the spread of a virus and this novel coronavirus is unfortunately no exception. In this case, a common myth is that it is some biological weapon, engineered in a laboratory as an agent of mass destruction. In the centre of this pandemic, another myth has been brewed and been used to sow seeds of confusion and denial in the minds of the uninformed. This theory is that the virus is an elaborate hoax created by the government for self-gratification and gain.

In Nigeria, the gulley between the government and the citizens has been so deepened following previous disappointments and deep-rooted issues. Therefore, there is no surprise that the government has been completely alienated from the people. There is a huge amount of mistrust for the government. Thus, some Nigerians have opined that the virus is propaganda serving as a ploy to attract funds from international and national donor agencies and then use the funds to corruptly enrich themselves and their cronies. Some influential and prominent religious leaders with millions of followers propagate and disseminate conspiracy theories claiming that COVID-19 is just fever/malaria or the effect of 5G network.

In addition, a fuel feeding the spread of this rumour has been the lack of disclosure of the identities of the confirmed cases. To the citizens that share this opinion, because they have not been personally affected by the virus neither have the victims been seen to be “real people”, the virus is a purely fictional character.

Taking root from this aforementioned line of reason, due to the only disclosed cases being public figures, it has been conjectured that there is a sort of selectiveness to the virus, with an insinuation that it is prejudiced against the elite [10]. Clearly this is inaccurate as although it may have seemed that the primary focus of the virus was on the crème de la crème of the country, with rapid speed it would extend beyond them to the impoverished. Which it has especially given the communal living that characterizes Nigerian living. Indeed, Nigeria has experienced widespread community transmission of COVID-19 virus since March 2020 [11].

## WAYS TO CURTAIL COVID-19 INFODEMICS IN NIGERIA

Infodemics as exemplified by the myths of immunity to the virus and that the disease is a hoax has very damaging and negative consequences to the prevention, containment, mitigation and treatment of COVID-19. Infodemics not only dilutes scientifically valid information and blurs the line between evidence-based facts and arm chair conjectures but also leads to the rejection, denial or outright indifference to evidenced based pharmaceutical and non-pharmaceutical interventions. There is overwhelming evidence that non pharmaceutical intervention (NPI) measures including lockdown, social distancing, hand washing and wearing of face masks breaks the virus transmission dynamics and flattens/depress the COVID-19 epicurve [12,13]. On the contrary, rejection of the NPI measures or haphazard adherence/ implementation of it (partly fuelled by COVID-19 misinformation/disinformation) results in surge in virus transmission with an attendant increase in morbidity and mortality. Thus, globally and in Nigeria, Covid-19 myths put lives at risk!

**Figure:**
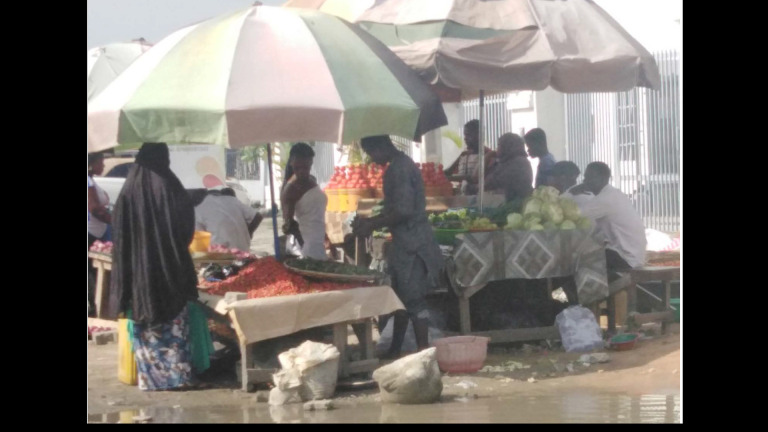
Photo: Vegetable and fruit open market in Lagos, Nigeria (from the author’s own collection, used with permission).

In the fight against this global pandemic, curtailing the tide of infodemics is as important as breaking virus transmission and spread. First and foremost, it is imperative that the public must be educated about misleading messages and be taught the skills to decipher rudimentary information. Questioning everything must be the norm. This is important in order to separate the fake news from the factual ones. In the long and short term, Nigeria education authorities should revise the school curriculum at all levels to emphasize critical thinking and logical reasoning.

Secondary, there must be control over the messages exposed on social media. Information sought out for guidance must be evidence-based and those shared must not only be informative but contain actionable behavioural change messages. In addition, scientific uncertainty pertaining to the virus infection biology and pathophysiology must be acknowledged and explained in a simple and focused manner. In combatting misleading messages, the NCDC must play a leading role, monitoring the information released at the federal, state and local levels and respond in real time to any misleading information. By collaborative works with stakeholders (particularly technology sectors and Nigeria Communication Commission), local groups, as well as international groups, censorship and other forms of regulations should be mandated to curb misinformation. Together these stated bodies can collaborate to quell incidences of fake news. NCDC should set up an actionable framework to reduce mix-messaging particularly the dichotomy between messages released by reliable sources like NCDC, WHO, etc., and politicians/government officials. It confuses the Nigerian public when messages issued by NCDC are contradicted by statements made by elected government officials or influential religious leaders. Most importantly, the COVID-19 response team in Nigeria especially NCDC, members of the presidential task force on COVID-19, ministers and governors must be seen to abide by the anti-COVID-19 mitigation measures. When these influential members of the Nigeria COVID-19 response team flagrantly and in public flout the mitigation measures on face masks, social distancing, etc., the public is left with the impression that the powerful and the privileged are above the “law”. Even more damaging from an epidemiological perspective is that the public consider the pandemic non-existent for if it does, our leaders should have adhered to the public health measures. Indeed, in Nigeria a new word has been coined for this behaviour and it is referred to “Audio” (Audio COVID-19 literally means a fictional pandemic existing only in the mind of Nigerian leaders).

In addition to all the above, Nigeria should immediately adopt and implement the framework for managing COVID-19 infodemic as detailed by WHO Technical Consultation on management of COVID-19 infodemics.
